# The Effect of the COVID-19 Pandemic on Grief Experiences of Bereaved Relatives: An Overview Review

**DOI:** 10.1177/00302228221143861

**Published:** 2022-12-01

**Authors:** Tamara van Schaik, Marije A. Brouwer, Nico E. Knibbe, Hanneke J. J. Knibbe, Saskia C. C. M. Teunissen

**Affiliations:** 1 Julius Center for Health Sciences and Primary Care, 8124 University Medical Center Utrecht, Utrecht, The Netherlands; 2 26093 LOCOmotion Research NL, Bennekom, The Netherlands

**Keywords:** bereavement, COVID-19, grief, health, pandemic

## Abstract

The COVID-19 pandemic has disrupted grief experiences of bereaved relatives and altered accustomed ways of coping with loss. To understand how bereaved relatives experienced grief during COVID-19, a review, using the overview method, was conducted. An overview of empirical data about this subject has been lacking and therefore, PubMed and CINAHL databases were searched for empirical studies published from January 1, 2020 until December 31, 2021. 28 articles were included in the review. Thematic analysis showed different emotional responses, changes in grief, the effect of absence during final moments, a lack of involvement in the caring process, the impact on communities and social support systems and the alteration of funerals among bereaved relatives. During COVID-19, death is characterized by poor bereavement outcomes and health implications, but bereaved also show signs of resilience and coping. Directions for future research about cultural and societal differences in grief and support methods are suggested.

## Introduction

The COVID-19 pandemic, which led to a worldwide health crisis and dramatic loss of human life around the globe, has provided unprecedented challenges to our lives, including the way people cope with loss and grief (Mayland et al., 2020; Stroebe & Schut, 2021). During this pandemic, an estimated 6.6 million people (Johns Hopkins, 2022)worldwide died because of COVID-19 and an even larger number (World Health Organization, 2022) died from other causes during the same period. The health risks and subsequent safety measures taken by governments to prevent the spread of COVID-19 have severely influenced the possibilities of bereaved relatives to say farewell to their loved ones, and deal with their grief (Stroebe & Schut, 2021).

Safety measures, including various lockdowns, visiting restrictions, social distancing measures and hygiene measurements were installed to prevent the further spread of COVID-19 and disrupted the social structures and rituals that were important aspects of coping with grief before the COVID-19 pandemic (Imber-Black, 2020). This influenced the way that relatives grieve the death of a loved one, no matter if the deceased died with or without being infected by COVID-19. Because grief emerges from relationships and interpersonal processes between the deceased and bereaved relatives (Jakoby, 2012), rituals are important as they mark the end of a life and provide us with powerful connections with others (Imber-Black, 2020). Funerals and other customs, that would normally help families, friends and communities to honor the deceased, provide support and share their grief, were impacted or sometimes even prohibited during COVID-19 (Burrell & Selman, 2020; Imber-Black, 2020; Walsh, 2020). Due to the range of safety measures, people have died without the presence of loved ones, often only surrounded by masked staff in hospitals (Sallnow et al., 2022). Furthermore, the social distancing measures not only affected the relationship between families and their dying loved ones, but also hindered bereaved relatives to turn to one another for support and comfort (Walsh, 2020).

How has this global health pandemic affected the grief of bereaved relatives? After 2 years of the pandemic, several studies on the effects of COVID-19 on grief of bereaved relatives are available. However, while there are some literature reviews on this topic, a comprehensive overview that includes empirical data has been lacking so far. Available literature reviews have either been published in the early stages of the pandemic, thus lacking empirical data (Stroebe & Schut, 2021), or focus on specific aspects of grief, such as the impact of funeral practices on bereavement during COVID-19 (Burrell & Selman, 2020), the impact of previous pandemics on grief and how that knowledge can be used during the COVID-19 pandemic (Mayland et al., 2020) or responses to bereavement support during human-made and natural disasters (Harrop et al., 2020). In order to understand how bereaved relatives experienced grief during the COVID-19 pandemic, we reviewed empirical studies about the impact on grief of bereaved relatives. These data provides in-depth knowledge about grief experiences emphasizing the needs of bereaved relatives and therefore, facilitate the right support methods. To fully encompass changes in grief experiences during COVID-19, we have focused on grief experiences after a death caused by COVID-19 as well as non-COVID-19 related deaths that took place during this period. To provide a comprehensive overview of grief experiences after the loss of a loved one during the COVID-19 pandemic, this paper focuses solely on the perspective of bereaved relatives themselves.

## Method

### Design

Overviews make a summary of the available literature in order to survey the literature and describe its characteristics. Overviews can provide a broad and comprehensive summation of a topic area and are, therefore, especially suitable for newer subjects, where the body of knowledge is still in development (Grant & Booth, 2009). We chose this method because the publications on COVID-related grief are still relatively new, and information on the topic is thus less defined. While quality assessments are sometimes included in Overview studies, it is not a necessary step. We decided not to perform a thorough quality assessment, since the quicker publication method of COVID-studies during the pandemic meant that there was limited information available to perform such an assessment accurately. Different analyses can be applied in Overview studies, for example thematic, conceptual of chronological. We chose a thematic analysis, as it best suited our research question.

### Theoretical Framework

The Dual Process Model of Coping with Bereavement (DPM) by Stroebe and Schut is used as theoretical framework (Stroebe & Schut, 2010), as according to this model grief is understood as a dynamic process of coping with loss. This model is especially suited to explain the more varied and dynamic ways in which bereaved relatives move back and forth in their process of grief, compared to existing phase-models of grief and bereavement where grief is described as a more unilateral process. The DPM provides several ways in which people come to terms with the loss of a loved one and, therefore, avoids generalizing the way grief affects individuals. The DPM defines two categories of stressors associated with grief. The first category is *loss-orientation*, which refers to a bereaved person’s focus on and dealing with stressful aspects of the loss experience itself. This includes the need for grief work, like visiting the grave or looking at photos of the deceased (Stroebe & Schut, 2021). A bereaved person can show a diversity of signs, ranging from denial, or the opposite, dwell on painful experiences (Stroebe & Schut, 2010). The second category is *restoration-orientation* and refers to other essential parts of grieving, like using sources of coping with stress (Stroebe & Schut, 2010, 2021). This includes, for example, reorientation on a life without the deceased person, searching for distraction from the grief (Stroebe & Schut, 2010) or performing practical activities such as taking up employment to compensate the deceased’s income (Stroebe & Schut, 2021). In the DPM model, the dynamic coping process is described as an oscillation between the two categories. A bereaved person moves back and forth between confronting and avoiding aspects of a loss. According to Stroebe and Schut, this oscillation is necessary for adaptive coping with grief (Stroebe & Schut, 2010).

In addition, the theory of Meaning Reconstruction is used to further understand the way people find meaning after losing a relative. The loss of a loved one is a stressful life event that often confronts bereaved relatives with questions related to meaning making, as their assumptions about life are shattered and they have to reorient on a life without the deceased. Meaning-making can be helpful in adaptation to bereavement in order to restore a sense of purpose and belonging (Neimeyer, 2011; Neimeyer et al., 2014). This theory could help understand the experiences of bereaved during COVID-19 in more detail. As the pandemic challenges meaning-making processes for almost all persons (Walsh, 2020), the sense-making of bereaved is especially challenged.

### Databases and Searches

A search string (see Table 1) was built by two authors (TS, MB) and reviewed by all authors. Subsequently, a search was performed in the databases PubMed and CINAHL. The search was refined to include peer-reviewed studies from January 1, 2020 until December 31, 2021. Articles that emerged from the searches were imported into Endnote, where duplicates were removed.

**Table 1.** Search Strategy.

Search Category	Corresponding search string
Grief	Grief OR bereave* OR grief* OR mourn* OR griev* OR funeral OR “death ritual”
Family	Family OR relative* OR famil* OR bereaved OR adults OR spouse* OR “next of kin”
COVID-19	COVID-19 OR SARS-CoV-2 OR COVID* OR lockdown OR “social distancing” OR pandemic OR corona*
Combined string	Grief OR bereave* OR grief* OR mourn* OR griev* OR funeral OR “death ritual” AND family OR relative* OR famil* OR bereaved OR adults OR spouse* OR “next of kin” AND COVID-19 OR SARS-CoV-2 OR COVID* OR lockdown OR “social distancing” OR pandemic OR corona*

### Study Selection

Articles were included if they fulfilled the following criteria: (1) a focus on grief experiences of relatives during COVID-19, (2) articles about death traditions, such as funerals, during COVID-19, (3) empirical research methods and (4) written in English. Studies were excluded when: (1) published before January 2020 and after December 2021, (2) focus on the experiences other than that of bereaved adolescents or adults, (3) concerning perinatal loss, (4) end-of-life care during COVID-19 without reporting about grief experiences of relatives and (5) concerning support methods for health care professionals. The researchers (TS, MB) made a first selection based on title and abstract. Articles were included if there was agreement about the title and abstract. In case of disagreement about suitability of articles, the researchers discussed their differing selections until agreement was reached on suitability for inclusion as full-text article. The eligible articles were then full-text screened by both researchers.

### Data Extraction

Data were extracted using a pre-designed form. Extracted data from the studies consisted of: author, year, country, aim, setting, study design, sample, the number of participants, method of data collection, the phase of COVID-19 during which the study was conducted and the central themes as described in the article. The data regarding relatives’ grief experiences during COVID-19 were then analyzed using a qualitative thematic approach. First the central themes were extracted from the articles. Subsequently, codes were added that summarized the meaning of the themes as described by the authors of a particular study. The codes described the main points that recurred throughout the data. Second, patterns were identified and the codes were combined into clustered themes. These themes were compared to the data, discussed by the researchers (TS, MB) and when agreement was reached, the themes were made final.

## Results

### Characteristics of Included Articles

**Figure 1.**
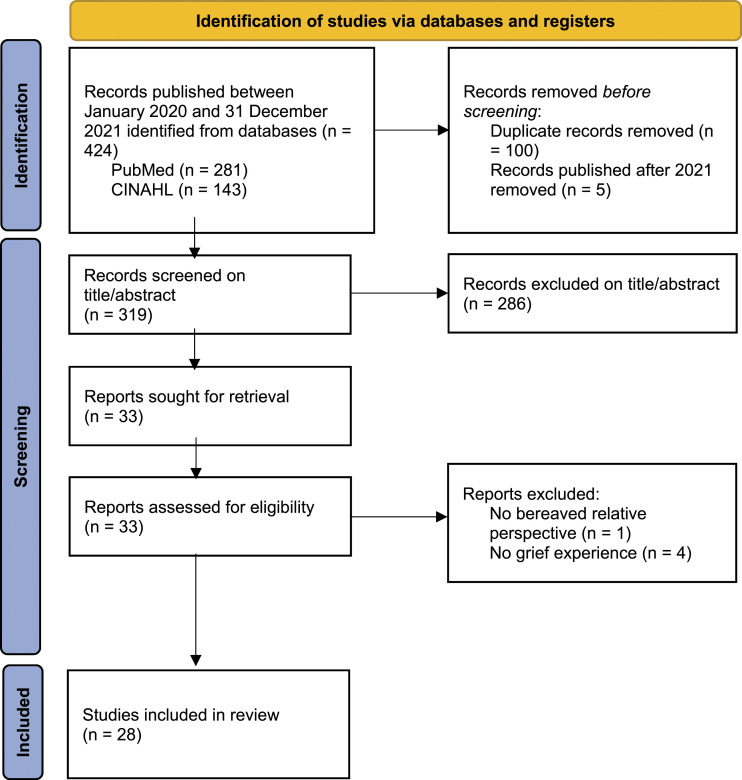
Flow diagram search strategy.

In total 424 studies were selected, 28 articles were included (see Figure 1).

Out of the total number of 28 studies, 16 articles reported qualitative data (Becqué et al., 2021; Borghi & Menichetti, 2021; Cardoso et al., 2020; De Leon Corona et al., 2021; Guité-Verret et al., 2021; Hamid & Jahangir, 2020; Helton et al., 2020; Hernández-Fernández & Meneses-Falcón, 2021; Kentish-Barnes et al., 2021; Menichetti Delor et al., 2021; Mohammadi et al., 2021; Moore et al., 2020; Mortazavi et al., 2021; Motamedzadeh et al., 2021; Selman et al., 2021; Testoni et al., 2021), 10 articles were quantitative studies (Carson et al., 2021; Chen & Tang, 2021; Eisma & Tamminga, 2020; Eisma et al., 2021; Lee et al., 2021; Schloesser et al., 2021; Şimşek Arslan & Buldukoğlu, 2021; Tang & Xiang, 2021; Wang et al., 2021), and two articles had a mixed-methods design (Harrop et al., 2021; Mayland et al., 2021). An overview of the included studies is provided in Table 2.

**Table 2.** Baseline Characteristics of Included Studies.

Author year	Country	Aim	Setting	Study design	Method of data collection	Sample	Number of participants studied	Phase of COVID-19 pandemic when conducting the study	Central themes as described in the article
Becqué et al. (2021)	NL	To give insight into aspects of end-of-life care practices that might have jeopardized or supported the dignity of the patients and their family members during the first wave of the COVID-19 pandemic in The Netherlands	Nursing home, hospital	Qualitative study	In-depth, semi-structured interviews	Bereaved relatives of people who died between March and July 2020 and resided in The Netherlands	Relatives *N* = 25	The COVID-19 pandemic in The Netherlands between March and July 2020	Dealing with an unknown illness
									Being isolated
									Restricted farewells
									Lack of attentiveness and communication
									Meaningful end-of- life moments
									Compassionate professional support
Borghi and Menichetti (2021)	Italy	To describe some spontaneous strategies that family members may adopt to cope with the loss of a relative for COVID-19	Not mentioned	Qualitative study	Written reports of calls and phone-related documents	Families of COVID-19 victims who died at the hospital	Family members *N* = 246	First wave of the pandemic in Milan, Italy	“Floating deaths”: Creating alternative rituals for grieving
									“Death in old age”: Normalizing a loss lived under extraordinary circumstances
									“Religious and existential anchors”: Addressing faith and hope
									“Away from the normal life”: More time to process the loss
									“Helpfulness”: Supporting others, supporting the self
									“Delivering the bad news”: Finding words, finding meanings
Cardoso et al. (2020)	Brazil	To understand the meanings individuals who have lost loved ones in this context assign to the phenomenon of suppressed funeral rituals	Not mentioned	Documentary research with a qualitative approach	Written personal documents, freely available to the public	Bereaved families who lost family members due to COVID-19	Family members *N* = 23	March, 1st till April, 20th. The beginning of the COVID-19 pandemic in Brazil	Unexpected, frightening, and invisible: Death closes its siege
					Reports of bereaved families available on the internet				Experiencing losses: There is no time to say goodbye, there is no closure
									The memory of the last hug: strategies to minimize suffering
Carson et al. (2021)	UK	To look at post-traumatic stress, coping skills and post-traumatic growth in relatives, who lost a loved one during the pandemic	Not mentioned	Quantitative study	Cross-sectional on-line questionnaire survey	Relatives who lost a close loved one due to COVID -19	Relatives *N* = 185	No specific period mentioned	94.6% of the participants scored 33 or more on the impact of event scale. This demonstrates that 9/10 people who lost a relative during the COVID -19 pandemic are experiencing post-traumatic stress disorder
									Respondents were most troubled by intrusive thoughts
									The main coping skills utilized by participants were linked to compassionate outreach
									Respondents reported most change in their appreciation of life and relating to others
Chen and Tang (2021)	China	To identify heterogeneous profiles of prolonged grief, post-traumatic stress and post-traumatic growth among people bereaved due to COVID-19 and to identify predictors of latent class membership	Not mentioned	Quantitative study	Online survey	Bereaved who lost a close person due to the COVID-19 pandemic	Bereaved *N* = 422	September and October 2020	Four profiles of prolonged grief, post-traumatic stress and post-traumatic growth were identified
									The relationship, both closeness and conflicts with the deceased were significant predictors of latent class membership
									The death of a younger person is more likely to cause a moderate-combined than a growth profile
									Time since bereavement and unexpectedness of the death were not significant predictors of latent class membership
De Leon Corona et al. (2021)	USA	To recognize those suffering from bereavement overload due to COVID-19.	Hospital	Qualitative study	Case study	3 cases encountered in health care system	Family member Patient Healthcare provider *N* = 3	No specific period mentioned	Complicated grief delays healing and worsens pain
									Palliative care must recognize and adapt to bereavement overload
									Bereavement overload can impact patients, family members and care givers
Eisma and Tamminga (2020)	NL	To test if grief severity is higher during than before the lockdown after non-COVID-19-related bereavement	Not mentioned	Quantitative study	Cross sectional survey	Bereaved adults who experienced a loss before or during the pandemic	Bereaved adults *N* = 1600	No specific period mentioned	Bereaved people who participated during the pandemic did not differ from bereaved people who participated before the pandemic
									People who became bereaved during the pandemic did not significantly differ from people who were recently bereaved before the pandemic
									Experiencing a recent loss during the pandemic did elicit higher levels of grief severity than experiencing a recent loss before the pandemic
Eisma et al. (2021)	NL	To investigate death characteristics (e.g., intensive care admission, unexpected death) and circumstances (e.g., secondary stressors, social isolation) during COVID-19, that supposedly will precipitate a worldwide increase of prolonged grief disorder (PGD) and persistent complex bereavement disorder (PCBD)	Not mentioned	Quantitative study	Self-report measures of demographic and loss-related characteristics and PGD and PCBD symptoms	People bereaved through COVID-19 (N = 49), natural causes (N = 1182), and unnatural causes (N = 210)	Bereaved *N* = 1441	No specific period mentioned	People who experienced COVID-19-related bereavement reported more severe grief than people who experienced natural losses (PGD d = 0.42; PCBD d = 0.35), but did not experience more severe grief than people bereaved through unnatural causes
									People who experienced COVID-19 related bereavement more often experienced the loss as unexpected than those experiencing natural loss
									People bereaved through COVID-19 were older and more often lost a parent and less often a child or friend than those experiencing unnatural loss
Guité-Verret et al. (2021)	Canada	To gain in-depth understanding of family caregivers' lived experiences of caregiving and bereavement in the context of the COVID-19 pandemic in Quebec, Canada. The study also aimed at providing new insight about caregiving and bereavement by analyzing the metaphors family caregivers use to report their experiences	Not mentioned	Interpretative phenomenological analysis	In-depth interviews	People who experienced the death of a loved one during the COVID-19 pandemic	Participants *N* = 20	First and second wave of the pandemic	Most important metaphors found: (1) being cut off from others, (2) living and facing obstructions and (3) feeling shockwaves
									According to the meaning of these metaphors, the participants appeared to be engaged in a search for (1) social connection, (2) narrative coherence and (3) recognition
Hamid and Jahangir (2020)	Kashmir	To examine the changing nature of death, dying and mourning among muslims of Kashmir due to the COVID-19 pandemic	Not mentioned	Qualitative study	Telephonic interviews, semi structured	Participants whose loved ones died after the outbreak of COVID-19	Participants *N* = 17	No specific period mentioned	Circumstances of death during COVID-19
									Dying in isolation
									Missing the last moments
									Changing rituals and practices surrounding death and dying
									Mourning in isolation
Han et al. (2021)	China	To learn from recent outbreaks of infectious diseases and further understand their impacts on bereavement	Not mentioned	Quantitative study	Online ecological recognition (OER) approach	Bereaved due to COVID-19 and due to non COVID-19 reasons	Bereaved *N* = 159	20 January 2020 to 1 March 2020	Emotional indices: CB people had significantly lower scores for negative emotional indices of psychological status in terms of stress (t (157) = 0.79, *p* = 0.162, d = −0.30) than NCB.
									Cognitive indices: CB people had significantly higher scores for cognitive indices of psychological status for collective behavior (t (157) = 1.92, *p* = 0.057, d = 0.37) but had significantly lower scores of cognitive indices of psychological status for life goals (t (157) = −1.86, *p* = 0.067, d = −0.31) than NCB.
Harrop et al. (2021)	UK	To investigate grief experiences, support needs and use of formal and informal bereavement support among people bereaved during the pandemic	Home	Mixed-methods	Longitudinal survey	Family or close friend bereaved since social distancing requirements were introduced in the UK	Bereaved *N* = 711	March to December 2020	Support needs and access to formal support
									Availability of (appropriate) support
									Knowledge and attitudes towards support use
									Accessing GP support
									Accessing support from friends and family
									Difficulties connecting and communicating with friends and family
									Lack of understanding and empathy
									Disrupted grieving
Helton et al. (2020)	USA	To examine ways in which COVID-19 has affected the bereavement experiences of parents whose children died of cancer before the pandemic	Not mentioned	Qualitative study	Semi-structured interviews	Parents of children who died of cancer	Parents *N* = 15	No specific period mentioned	Change in support
									No effect on grief
									Familiarity with uncertainty/ability to cope
									Change in contact with care/research team
									Compounded isolation
									Thankfulness
									Trigger
									Forced pause/time to grieve
									Loss of routine or personal time
									Additional stress
									Constant reminders/no break from grief
Hernández-Fernández and Meneses-Falcón (2021)	Spain	To (a) describe the experience of the loss of a loved one without the culturally determined rituals for the farewell, (b) explore how the grief process experienced by family members is initiated under the conditions generated by the pandemic and (c) study the existence of factors complicating grief that are associated with this type of loss	Mixture of settings	Qualitative study	In depth interviews	First or second- degree relatives of a deceased person, hospital employees, senior residence employees, funeral services professionals, emergency services professionals, social workers, firefighters and chaplains	Participants *N* = 48	July to November 2020	Goodbyes
									Grief
									Emotions
									Moment of death
									Spirituality
									Informing family member
									Unexpected death
									Deceased’s belongings
									Healthcare and mortuary
									Protocols
									Rites
									Sitting vigil with the corpse or ashes
Kentish-Barnes et al. (2021)	France	To better understand the experiences of bereaved family members of patients who died in an ICU during the COVID-19 pandemic, from the time of hospital admission until after the patient’s death	Hospital	Qualitative study	Semi structured in depth interviews	Bereaved family members	Bereaved *N* = 19	June to September 2020	Difficulty in building a distance relationship with the ICU clinicians and the experience of solitude of the patient in the ICU and the risks of separation
									Disrupted end-of-life rituals and the feeling of “stolen moments” with the deceased
Lee et al. (2021)	USA	To examine the incremental validity of the PGS in identifying mourners at risk of harmful outcomes	Not mentioned	Quantitative study	Cross sectional survey	People bereaved through COVID-19	Bereaved *N* = 1065	November 2020	Participants scored above cut score of >7 on the PGS (56,6%)
									Participants coped with their loss using drugs of alcohol for at least several days or 2 weeks (69,7%)
									PGS scores were not associated with time since loss
									The PGS explained variance in functional impairment, meaning-making difficulties, and substance use coping
Mayland et al. (2021)	UK	To explore bereaved relatives' experiences of quality of care and family support provided during the last days of life; to identify the impact of factors associated with perceived support	Mixture of care settings	Mixed-methods	Observational online survey, with thematic analyses of free-text responses	Individuals (≥18 years) who had experienced the death of a relative/friend (all care settings) within the United Kingdom during the COVID-19 pandemic	Respondents *N* = 278	June to September 2020	Public health restrictions compounding the distress of ‘not knowing’
									Disparate views about support from doctors and nurses
									Challenges in communication and level of preparedness for the death
									Delivery of compassionate care
									Emotional needs and potential impact on grief
Menichetti Delor et al. (2021)	Italy/Norway	To explore the families' experiences and needs collected during these calls, and the role that the psychologists played through the call	Hospital	Qualitative study	Phone-related documents review, observation of psychologists’ peer group discussions and semi-structured interviews	Bereaved families due to COVID-19	Bereaved family members *N* = 246	No specific period mentioned	Experiences
									Without death rituals
									Solidarity
									Unexpected and fast
									Unfair
									Unsafe
									Coexistent with other stressors
									Needs
									To give meaning
									To express emotions
									To say the last goodbye
									To remember
									To solve practical issues
Mohammad et al. (2021)	Iran	To investigate the psychological challenges and issues which the families of COVID-19 victims are faced with. The present study aims to identify the mental health crises which the families of COVID-19 deceased victims are going through	Not mentioned	Qualitative study	Semi-structured interviews	Families of COVID-19 victims	Family members *N* = 16	February to May 2020	Emotional shock
									Feelings of guilt and rumination
									Bitter farewell
									Strange burial
									Concern about unreligious burial
									Fear of the future
									Instability in the family stigmatization and complications in social interactions
									Lack of job security and difficult financial conditions
Moore et al. (2020)	USA	To discuss COVID-19 relative to black people and their overrepresentation among those who are infected and died from the disease	Not mentioned	Qualitative study	Case study	Relatives of COVID-19 victims within the black community	Respondents *N* = 4	No specific period mentioned	The economic impact on black people in this country as a result of COVID-19 will no doubt be generational
									Due to the beliefs and rituals that are cultural bedrocks as related to death, dying, and funeral practices embraced by many members of the black community; suffering the COVID-19 viral infection and potentially dying alone, is an excruciating experience for both patients and their families
Mortazavi et al. (2021)	Iran	To gain a deep understanding of the experience of mourning during COVID-19 pandemic by exploring the experiences of survivors of the death of their loved	Not mentioned	Qualitative study	Semi-structured interviews	Iranian citizens, all muslims, who lost a family member from the corona virus disease	Participants *N* = 15	No specific period mentioned	Ambiguity and desperation
									Incoherent narrative
									Feeling lonely
									The conflict between fear of and need
									Becoming relieved by alternatives
Motamedzadeh et al. (2021)	Iran	To explain the experience of bereaved families of patients with COVID-19	Not mentioned	Qualitative study	Semi-structured interviews	Bereaved families of patients with COVID-19	Participants *N* = 20	August 22, 2020 to May 21, 2021	Adaptation to the new world
									Psychological symptoms
									Role conflict
									Miracle of belief and faith
Schloesser et al. (2021)	Germany	To describe the experiences of bereaved relatives of patients who died during the SARS-CoV2 pandemic, regardless of whether patients were infected with SARS-CoV2 or not	Hospital	Quantitative study (thematic analyses of free text responses)	Online survey	Bereaved relatives of patients with and without an SARS-Cov2 infection	Bereaved relatives *N* = 81	First peak of the pandemic	Visiting restrictions and end-of-life companionship during the SARS-CoV2 pandemic
									Burdens by visiting restrictions
									Impact of the visiting ban on visiting opportunities
									Place of death
									SARS-CoV2 infection
									Online communication with the dying patient
									Bereaved relatives‘ perspective on communication with the healthcare team during the SARS-CoV2 pandemic
Selman et al. (2021)	UK	To explore the views and experiences of twitter social media users who reported that a relative, friend or acquaintance died of COVID-19 without a family member/friend present	Not mentioned	Qualitative study	Thematic analyses of twitter data	Twitter posts mentioning a relative, friend or acquaintance who had died alone of COVID-19	Twitter posts *N* = 196	7th–20th April 2020	Restrictions
									End of life
									Emotional impact
									Disrupted bereavement
									Explicit function of tweet
Şimşek Arslan and Buldukoğlu (2021)	Turkey	To examine the grief rituals and grief reactions of individuals who experienced the death of a loved one during the COVID-19 pandemic	Not mentioned	Quantitative study	Online survey	Individuals who experienced the death of a loved one during COVID-19	Participants *N* = 114	October 15, 2020 to January 15, 2021	The majority of participants (81.6%) stated that the COVID-19 pandemic affected the grieving process
									The participants who stated that the COVID-19 pandemic affected the grieving process showed more physiological grief reactions
									The implementation of grief rituals did not affect the grief reactions (*p* > .05)
Tang and Xiang (2021)	China	To estimate the prevalence of PGD and investigated demographic and loss-related factors associated with prolonged grief symptoms among Chinese individuals bereaved due to COVID-19	Not mentioned	Quantitative study	Online survey	Individuals who lost a close person due to COVID-19	Participants *N* = 422	September 1, 2020 to October, 3 2020	No difference was found in levels of grief symptoms between participants whose close one died more than 6 months ago and those who experienced the loss less than 6 months ago
									More severe prolonged grief symptoms was associated with losing a close person by COVID-19 rather than complications (B: 5.35; 95% CI: 0.54–10.05), losing a partner (B: 7.80; 95% CI: 3.24–12.37), child (B: 8.15; 95% CI: 1.03–15.26), and parent (B: 5.49; 95% CI: 1.49–9.48) rather than losing a relative or a person with other relationship, feeling more traumatic about the loss (B: 1.71; 95% CI: 0.52–2.90), being closer with the deceased (B: 1.60; 95% CI: 0.34–2.86). Moreover, losing a grandparent (B: 6.62; 95% CI: 0.53–12.71) and having more conflicts with the deceased (B: 1.05; 95% CI: −0.008–2.11) were related to higher levels of grief symptoms assessed by TGI-SR.
Testoni et al. (2021)	Italy	To examine bereavement experiences among family members, how they processed their grief, and how they used social networks in particular by uploading photographs during the working-through of bereavement	Not mentioned	Qualitative study	Interviews	Individuals who lost a loved one because of COVID-19	Participants *N* = 40	First wave of the pandemic	Abandonment: Anger and guilt
									Dehumanized disappeared
									Derealization and constant rumination
									Social support and the importance of sharing photos on facebook
Wang et al. (2021)	USA	To examine if COVID-19 bereavement corresponds with older adults’ reporting depression in 27 countries and test for variations by gender and country context	Not mentioned	Quantitative study	Data collected from survey of health	51,383 older adults (age 50–104) living in 27 countries, of whom 1363 reported the death of a relative or friend from COVID-19	Respondents *N* = 51,383	June to August 2020	COVID-19 bereavement is associated with significantly higher probabilities of both reporting depression and reporting worsened depression among older adults
									Net of one’s own personal loss, living in a country with the highest COVID-19 mortality rate is associated with women’s reports of worsened depression but not men’s
									The country’s COVID-19 mortality rate does not moderate associations between COVID-19 bereavement and depression

The included articles covered a wide range of themes related to the way bereaved families and friends dealt with grief during the COVID-19 pandemic and how grief rituals, grief responses and grief severity have changed. Our analysis yielded insights into seven different themes: (1) the emotional impact on grief, (2) how grief has changed, (3) being absent or present during the final moments, (4) lack of involvement in the caring process (5) social dimension of grief, (6) changing rituals, (7) finding meaning in a difficult time.

#### The Emotional Impact of the COVID-19 Pandemic on Grief

Eight studies reported on the emotional impact of COVID-19 on the grief of bereaved relatives (Becqué et al., 2021; Cardoso et al., 2020; Hamid & Jahangir, 2020; Hernández-Fernández & Meneses-Falcón, 2021; Menichetti Delor et al., 2021; Mohammadi et al., 2021; Mortazavi et al., 2021; Selman et al., 2021). They report experiences of fear and uncertainty, sadness and despair, which were sometimes accompanied with anger and frustration about healthcare policies or people who were not following the lockdown regulations. The grief reactions of bereaved relatives mentioned by different recent studies show similarities between experiencing a loss during the COVID-19 pandemic and grief after a traumatic loss. Relatives experienced increased levels of grief severity, and increased rates of Prolonged Grief Disorders compared to before the COVID-19 pandemic (Carson et al., 2021; Eisma & Tamminga, 2020; Lee et al., 2021; Şimşek Arslan & Buldukoğlu, 2021; Tang & Xiang, 2021). Mayland et al. (2021) report that relatives described their experiences as ‘traumatic’, as some had to deal with several losses simultaneously (Menichetti Delor et al., 2021). Carson et al. (2021) found that 90% of participants in their study who lost a relative during the COVID-19 pandemic experienced symptoms equating to a post-traumatic stress disorder.

Cheng and Tang (2021) describe how, due to the constant media-attention to COVID-19 related loss, the period after the moment of death did not bring peace, but brought an ever-increasing exposure to death, constantly reminding bereaved relatives of their loss, thus mitigating the healing effects of passing time. This might lead to a longer duration of acute grief (Tang & Xiang, 2021). Relatives were also often surprised by the rapid progress of the disease and sudden death, provoking a sense of disbelief (Becqué et al., 2021; Eisma et al., 2021; Hernández-Fernández & Meneses-Falcón, 2021). The shock of a sudden deterioration of the health of a loved one left relatives without time to prepare themselves for the moment of death, or to say goodbye (Mayland et al., 2021; Menichetti Delor et al., 2021; Mohammadi et al., 2021). Finally, bereaved relatives reported feelings of guilt, associated with fears of having transmitted COVID-19 to their family members and therefore, causing their death (Mohammadi et al., 2021). The deceased did not die with dignity, in the views of the relatives, because cultural or religious practices could not be performed (Hamid & Jahangir, 2020).

#### How COVID-19 has Changed Grief

Grieving processes changed as a result of the COVID-19 pandemic. Physical absence dominated and sometimes grieving relatives were physically separated from the body of the deceased. Hernández-Fernández and Meneses-Falcón (2021) compared the effect that the COVID-19 pandemic had on grief to the grief that generally follows after cases of missing persons or missing bodies, causing possible difficulties for relatives in accepting the reality of the loss. Sometimes relatives felt angry about being kept away from the dying person and their body after the moment of death during the pandemic, which they described as a dehumanizing experience (Kentish-Barnes et al., 2021). Testoni et al. (2021) mention the impossibility to accompany a dying person, which can result in feelings of being ‘detached from the world’ and relatives searching for tangible evidence of the loss.

With respect to how COVID-19 has changed grief, some actual studies distinguished between the impact of a death caused by COVID-19 and when loved ones died of other causes. Differences between COVID-19 related grief and non-COVID-19 related grief are mentioned (Chen & Tang, 2021; Eisma & Tamminga, 2020; Han et al., 2021; Motamedzadeh et al., 2021; Schloesser et al., 2021). First, people who experienced COVID-19 related grief report more severe grief than people who grieved because of natural losses (Eisma et al., 2021). The experience of natural death is found to be different as compared to a death caused by COVID-19, because death due to COVID-19 occurs in the majority of cases unexpectedly (Motamedzadeh et al., 2021). Therefore, relatives of patients with COVID-19 felt more burdened by the pandemic compared to those suffering from other illnesses (Schloesser et al., 2021). Additionally, negative emotions of people bereaved by COVID-19 were found worse than for non-COVID-19 bereaved (Han et al., 2021). COVID-19 grief even corresponds with self-reported worsened depression (Wang et al., 2021). Finally, COVID-19 bereaved relatives had more difficulty recovering from the death of a loved one and were less able to set goals for the future (Han et al., 2021).

A final characteristic related to how COVID-19 has changed grief concerns the influence of cultural and societal differences surrounding death. Some studies, for example, the ones conducted in Kashmir and Iran, report stigmatization followed by a COVID-19 related loss (Hamid & Jahangir, 2020; Mohammadi et al., 2021). In some communities people were terrified of interacting with bereaved families and stayed away from them because they feared getting infected (Mohammadi et al., 2021). As a result, bereaved families experience complications in social interactions and finding support (Hamid & Jahangir, 2020). Especially when the deceased is the father, the family situation can become instable because some cultures do not allow mothers to keep their children if they want to live independently or get married again (Mohammadi et al., 2021).

#### Being Absent or Present During the Final Moments

Being absent at the moment of death was reported as a devastating burden on grieving relatives and affected their well-being on long term (Hamid & Jahangir, 2020; Menichetti Delor et al., 2021; Selman et al., 2021). Visiting options were often severely restricted or even completely prohibited, thus limiting options for relatives to be near their loved ones during the last phase of their lives. Regulations sometimes even made it impossible for relatives to be physically present or touch them, which affected their wellbeing (Becqué et al., 2021; Cardoso et al., 2020; Hamid & Jahangir, 2020; Mayland et al., 2021; Mortazavi et al., 2021). Some relatives said goodbye over the phone or through windows (Becqué et al., 2021; Selman et al., 2021). In some cases it was not so much the case that the regulations made the presence impossible, but relatives themselves chose not to visit their dying loved one because they feared getting infected (Becqué et al., 2021; Hernández-Fernández & Meneses-Falcón, 2021; Mortazavi et al., 2021). This sometimes lead to a dichotomy between the need for physical presence with their loved one, and the fear of COVID-19 (Mortazavi et al., 2021).

Physical proximity was no longer a given fact and therefore, the importance of being present during the final moments, and the effect it had on grief when the option was not available, became very visible during the pandemic. Relatives experienced guilt and feelings as if they had abandoned the dying person, because their loved ones died in isolation (Hamid & Jahangir, 2020; Kentish-Barnes et al., 2021; Moore et al., 2020). Not being able to be present at the time of death severely affected the meaning bereaved relatives attributed to the death of their loved one and were a major factor in pandemic-related stress among relatives (Schloesser et al., 2021). They experienced their farewell as incomplete, because they were not allowed to touch or get close to their loved one (Hernández-Fernández & Meneses-Falcón, 2021), which in some cases lead to a denial of accepting the loss (Hamid & Jahangir, 2020).

Not letting someone die alone was found to be essential for relatives and it helped them with finding closure if visitation was made possible (Kentish-Barnes et al., 2021). Although physical presence was impaired by measures such as social distancing, facemasks and the inability to touch each other (Becqué et al., 2021), the presence of social support, also if not physical, was found meaningful (Menichetti Delor et al., 2021). Extended visiting opportunities in the dying phase were appreciated by relatives (Becqué et al., 2021), although some relatives felt these opportunities were offered too late because their loved ones were no longer responsive at that moment (Schloesser et al., 2021).

#### Lack of Involvement in the Caring Process

Social distancing measures had consequences for the involvement of relatives in the care for their loved one in the terminal phase. Because they could no longer be physically present, one of the challenges relatives faced was a lack of communication and information about, for example, prognosis and decisions that were made by the medical staff. Some relatives felt frustrated and angry by not receiving essential information about their dying family member, were not provided with sufficient time to say their goodbyes, and in some cases, were even not informed about the death of their loved one on time (Mayland et al., 2021; Menichetti Delor et al., 2021). Being unable to comfort a sick loved one and participate in health care choices, affected the way relatives coped with the death (Moore et al., 2020).

Reports on psychosocial support of relatives by the medical staff were mixed. Some relatives felt that their own psychosocial needs were neglected by the healthcare team (Mayland et al., 2021), and in some cases, relatives did not have any contact with the healthcare team at all after the death of their loved one (Schloesser et al., 2021). On the other hand, several studies showed that bereaved relatives assessed the support as good or excellent and felt treated empathically (Becqué et al., 2021; Schloesser et al., 2021). Relatives appreciated the extra efforts made by nurses to take care of their loved one in their absence, for example arranging window visits or phone calls. As a result, relatives felt emotionally understood and perceived open and clear communication as helpful (Becqué et al., 2021).

#### Social Dimension of Grief

During their grief, relatives emphasized a need for physical contact with family and friends (Harrop et al., 2021), as well as a need for human connection (Guité-Verret et al., 2021); to be close to each other and share the burden of grief, grieve together and remember the loved one together (Menichetti Delor et al., 2021). However, the COVID-19 pandemic disrupted this need for human connection, and caused relatives to become isolated from each other. As a result relatives experienced an increased sense of isolation (Helton et al., 2020), loneliness (Mortazavi et al., 2021), and a feeling of being “cut off” from others (Guité-Verret et al., 2021). Not being able to share their grief could lead to a lack of understanding from friends and family, causing bereaved relatives to feel alienated (Harrop et al., 2021). Restrictions that impeded social mourning practices and family gatherings meant that the bereaved were visited less often, which resulted in them being left to mourn alone (Hamid & Jahangir, 2020).

While studies showed that bereaved relatives emphasized their need for a human connection, they also found it difficult to connect with others during their grief (Guité-Verret et al., 2021; Harrop et al., 2021). They sometimes felt torn between their need for physical presence and the fear of being affected by the virus (Mortazavi et al., 2021) and, therefore, felt a lack of physical, emotional and spiritual connection with others (Guité-Verret et al., 2021). Despite sharing their grief with other family members of the deceased patient, bereaved relatives described their grief as a solitary process. Due to the restricted options to visit dying family members, families were separated during their time of grief (Becqué et al., 2021; Kentish-Barnes et al., 2021).

The isolation that relatives felt during their process of grief emphasized their need for togetherness (Menichetti Delor et al., 2021). In the absence of social gatherings they searched for other ways to share their grief. Relatives mentioned the importance of support suited for their specific culture or subgroup (Harrop et al., 2021). Telephone or web-based support, such as the use of social media platforms, were found helpful to both seek out for and offer support and remember the life of their loved ones (Harrop et al., 2021; Testoni et al., 2021). By being able to connect, share grief and support others, relatives fostered their sense of helpfulness, power and self-confidence (Borghi & Menichetti, 2021).

#### Changing Rituals

Performing rituals is an important aspect of grief. During the COVID-19 pandemic, relatives valued the rituals that facilitated adjustment to their grief after the death of a loved one (Hernández-Fernández & Meneses-Falcón, 2021; Kentish-Barnes et al., 2021). The way that the COVID-19 pandemic affected the performance of rituals varied. Bereaved families in some countries were able to perform (smaller) rituals in person, while in other cases social rituals after death were severely limited or completely prohibited. Bereaved relatives who were able to perform some ritual act afterwards recognized the relief that the commemoration brought them (Hernández-Fernández & Meneses-Falcón, 2021; Kentish-Barnes et al., 2021). Carson et al. (2021) reported that contentment about funeral arrangements promoted post-traumatic growth after a loss.

COVID-19 disrupted many families in their possibilities to perform meaningful rituals at their time of grief. Many relatives were not able to attend funerals or perform grief rituals, only a small number of families were able to organize a ceremony up to their expectations (Cardoso et al., 2020; Kentish-Barnes et al., 2021; Şimşek Arslan & Buldukoğlu, 2021). Studies found that the impossibility of holding a mourning ceremony in the previous traditional form made it harder to bear the loss of a loved one (Motamedzadeh et al., 2021; Testoni et al., 2021). Participants reported that the absence of customs and rituals made the death of the deceased unbelievable and the relatives’ loss to be incoherent (Mortazavi et al., 2021; Şimşek Arslan & Buldukoğlu, 2021). They felt that a stage in their grieving process was skipped (Cardoso et al., 2020) resulting in a ‘floating death’, in which death remained disembodied and unreal (Borghi & Menichetti, 2021; Kentish-Barnes et al., 2021; Mohammadi et al., 2021). Respondents also reported that being unable to perform their rituals resulted in feelings of loneliness (Mortazavi et al., 2021), depression, exclusion (Hamid & Jahangir, 2020) and guilt towards the deceased (Kentish-Barnes et al., 2021). Especially in communities that heavily relied on a sense of community to ease the burden of death and loss, the effects of not being able to perform rituals were disproportionately felt (Moore et al., 2020), as social support and collective mourning was made impossible (Harrop et al., 2021; Menichetti Delor et al., 2021; Şimşek Arslan & Buldukoğlu, 2021). For religious families, not being able to perform religious-cultural rituals was a severe burden (Hamid & Jahangir, 2020). For some, one of the worst crises after the death of a loved one was the concern that family members would be buried in an unorthodox or unreligious manner (Mohammadi et al., 2021). Social distancing disrupts these traditional religious grieving practices (Moore et al., 2020) and therefore, a lack of emotional support was also experienced (Mortazavi et al., 2021). For some, activities such as praying and averting the mind in lonely times are helpful depending on the culture and beliefs (Motamedzadeh et al., 2021).

On the other hand, while the COVID-19 pandemic caused a disruption in the way that rituals could be carried out, it also gave families a chance to re-evaluate the rituals they knew, or find new ways to say goodbye to their loved ones (Kentish-Barnes et al., 2021). Without the familiar funeral rituals, out of necessity families turned to alternatives to sooth themselves and create a sense of community (Menichetti Delor et al., 2021; Mortazavi et al., 2021). Overall, modern technologies - phone calls, video calls, messages – were rediscovered and played a key role to help families not feeling segregated and alone. Online meetings made it possible for family members to virtually be at the bedside (Borghi & Menichetti, 2021; Moore et al., 2020), and online social networks were used as a platform to share grief and loss (Mortazavi et al., 2021), although for some these alternatives lacked authenticity and meaning (Cardoso et al., 2020; Kentish-Barnes et al., 2021; Mortazavi et al., 2021).

#### Finding Meaning in a Difficult Time: Positive Effects of the COVID-19 Pandemic on Grief

Although the COVID-19 pandemic and the following restrictions were initially perceived as a burden, the effect of the pandemic varied per individual. While some relatives found their grief to be exacerbated by the isolation and uncertainty of the COVID-19 restrictions, others found strength or recognized their resilience in their grief (Helton et al., 2020). In addition, the social isolation that followed from the COVID-19 restrictions was beneficial for some bereaved relatives. It provided them with time to process their loss and a break from normality to work on their grieving (Borghi & Menichetti, 2021; Helton et al., 2020). This gave them time to reflect on their loss and emotions, facilitating their grieving process, often in small social bubbles together with their closest family members (Helton et al., 2020).

The pandemic also stimulated finding alternative ways of being present at the last phase of patient’s lives, which helped next of kin to accept the situation (Cardoso et al., 2020; Kentish-Barnes et al., 2021). Talking about death, while difficult, helped bereaved relatives to process the loss of their loved one (Borghi & Menichetti, 2021). Navigating through the continuously changing COVID-19 restrictions sometimes helped families as a diversion from the painful reality of death (Menichetti Delor et al., 2021).

Relatives described several ways in which they gave meaning to the death of their loved one during these extraordinary circumstances, which helped to cope with their loss and grief. For some, faith and spirituality provided stability and “inner anchorage” during the uncertainty and unpredictability of the pandemic (Borghi & Menichetti, 2021; Menichetti Delor et al., 2021). Others found comfort in the notion of a ‘greater purpose’ in the loss of their loved one (Cardoso et al., 2020), or in normalizing their loss as something that could not be avoided, or that was destined to happen anyway (Borghi & Menichetti, 2021; Menichetti Delor et al., 2021).

## Discussion

This review provides an overview of the various ways in which the COVID-19 pandemic and the subsequent safety measures have impacted the grieving process of bereaved relatives. The 28 studies included in this overview show that the COVID-19 pandemic has drastically altered the way in which bereaved relatives experience and express their grief, and they describe both negative and positive outcomes. Because of the diversity of countries represented in the study, different grief reactions are mentioned, depending on the situation in a specific country, the phase of the pandemic when conducting the study and cultural differences in how people cope with grief. Despite this diversity the seven themes in which our findings are presented show similarities in how bereaved relatives experience grief and what matters as being meaningful when suffering from the loss of a loved one on an individual, social and cultural level.

On an individual level, COVID-19 influenced accustomed ways of coping with grief, by impacting opportunities to say goodbye to a loved one, familiar death rituals, social gatherings and, therefore, social support systems. Through these changes, it became apparent that COVID-19 changed the narrative of coping with grief, and the way that relatives dealt with their emotional response to a loss. Because the pandemic and the accompanying safety regulations hindered the ways that relatives could be close to their loved ones at the last phase of their lives, the moment of separation was not the moment of death, but the moment a person was admitted to the hospital (Hernández-Fernández & Meneses-Falcón, 2021). Farewells that have taken place were quick and mostly without physical contact or awareness that it would be the last time relatives would see one another (Becqué et al., 2021; Hernández-Fernández & Meneses-Falcón, 2021). Sometimes, this emotional impact resulted in increased levels of grief severity, grief disorders (Carson et al., 2021; Eisma & Tamminga, 2020; Lee et al., 2021; Şimşek Arslan & Buldukoğlu, 2021; Tang & Xiang, 2021) or traumatic experiences (Mayland et al., 2021).

Social distancing measures, which caused a lack of involvement in caring for a dying person and an inability to be with a loved one at the moment of death show the importance of the social dimension of grief. Grief can be understood as a social emotion emerging from relationships with others (Jakoby, 2012), thus posing difficulties for the grieving process if bereaved relatives are not able to share their grief with others. The absence of physical contact (Harrop et al., 2021) and social support, resulting in feelings of isolation and loneliness, further emphasize this need for human connection in times of grief (Guité-Verret et al., 2021; Hamid & Jahangir, 2020; Helton et al., 2020; Mortazavi et al., 2021). The lack of social support is sometimes characterized by stigmatization (Mohammadi et al., 2021), as in some countries people hide their illness and, therefore, do not seek medical care or the support of others (Hamid & Jahangir, 2020).

Finally, the COVID-19 pandemic changed grief on a cultural level, by impacting rituals and cultural practices. The burden of the restrictions for relatives is influenced by societal and cultural differences. Especially for religious families and countries with a strong sense of community (Hamid & Jahangir, 2020; Mohammadi et al., 2021; Moore et al., 2020), relatives suffered from the absence of collective mourning (Şimşek Arslan & Buldukoğlu, 2021). However, studies also report creative ways in which relatives cope with the restrictions, such as online meetings and the livestreaming of funerals (Borghi & Menichetti, 2021; Moore et al., 2020; Mortazavi et al., 2021; Stroebe & Schut, 2021). Literature argues that in coming together, families and communities are able to endure suffering and loss and sometimes even grow stronger (Walsh, 2020). Thus, this review not only shows the hardship bereaved relatives encountered during the COVID-19 pandemic, it also emphasizes the resilience in people and their ability to find meaning during stressful life events (Walsh, 2020).

These findings can further be understood in the context of the Dual Process Model (DPM) by Stroebe and Schut (Stroebe & Schut, 2010). With regard to the dynamic process of grief, as described in the DPM, we have found that bereaved relatives move back and forth between orientation on the loss itself and elements of restoration by finding new ways to come to terms with the loss of a loved one as their usual support systems were impacted. On the one hand restrictions on farewells can cause feelings of denial and problems with accepting the death of a loved one (Hamid & Jahangir, 2020; Schloesser et al., 2021). However, bereaved relatives showed adaptation to these circumstances as the findings emphasize the need for grief work when dealing with stressful aspects of the loss. Relatives actively sought and found alternatives to express their grief. Elements of restoration were impacted during the pandemic, because bereaved relatives were constantly reminded of their loss by the media attention about COVID-19 (Chen & Tang, 2021), which made it hard to find distraction from their grief (Stroebe & Schut, 2010). In addition, the results suggest that in some cases relatives found it difficult to recover from the loss and were less able to set goals for the future (Han et al., 2021), which impacts the ability to reorient on a life without the deceased (Stroebe & Schut, 2010).

Reorientation after a loss often includes meaning-making. According to literature, processes of grief occur on different levels, personal, inter-personal and social levels (Neimeyer et al., 2014). To establish the meaning of a deceased’s life and death, bereaved relatives are both in need of private and public moments to express their grief. Therefore, a bereaved individual also relies on the significance of the loss in the wider community and the meaning attributed to it by family and friends (Neimeyer et al., 2014). Especially this part of grief was affected by the restrictions during COVID-19. Bereaved could not always be with their dying loved one and were separated from others. Besides, important rituals that would normally help construct the meaning of a loss within the community were impacted. Thus, in times of a pandemic where the social structures around death and dying are changing, bereaved relatives are faced with additional challenges that force them to integrate the loss of a loved one within their life on their own.

Despite the negative effect on different aspects of coping with grief, the COVID-19 restrictions also had its benefits. Isolation measures provided relatives with a break from obligations, giving them time to process their loss on their own terms (Borghi & Menichetti, 2021; Helton et al., 2020). Other relatives found solace in their faith, which helped them deal with the uncertain times of the pandemic (Borghi & Menichetti, 2021; Menichetti Delor et al., 2021). Alternatives to create a sense of community and perform rituals together were also found. These examples, where bereaved actively tried to connect with others, show that establishing meaning after a loss is often a social process and that interactive processes can support individuals in their quest for meaning-making (Neimeyer et al., 2014). Consequently, this review shows that bereaved relatives are able to navigate through the buildup of stressors given by the circumstances of the COVID-19 pandemic and, therefore, indicate that it is still possible to find a balance in their grieving process by oscillating between an orientation towards aspects of the loss and elements of restoration (Stroebe & Schut, 2010).

## Limitations and Strengths

This review is the first in which an overview of empirical studies regarding the impact of COVID-19 on the grief of bereaved relatives is provided. The overview methodology has been helpful in reviewing the topic of grief in times of COVID-19 and enables to approach grief from a broad perspective. The dual process model of coping with bereavement by Stroebe and Schut helped to select paradoxal themes as reflection of dynamic grieving processes.

This review has its limitations, as it only included studies with a publication date until January 1, 2022. Since the COVID-19 pandemic is not over yet, new studies are being published which might give new or improved perspectives on the topic. Furthermore, we have focused on grief in adults and adolescents. Experiences about how the pandemic influenced grief in, for example, children or cases of perinatal loss, were not included. In addition, perspectives of grief other than that of bereaved relatives were excluded, so the data is not able to provide information on grief of, for example, healthcare professionals or other members of society.

The study also has some cultural limitations. Although some cultural variation is represented in this review, the majority of studies were conducted in Europe and North-America. Studies from Asia and the Middle East are included but might be underrepresented. Further research might study cultural differentiations in more detail. Finally, the pandemic and its consequences have changed in the course of the past 2 years. Treatment and preventive options have changed considerably. It is possible that the different stages of the pandemic are of influence as well. Long-term follow up of studies published may provide more insight in the effect of different stages of the pandemic.

## Conclusion

This review provides an overview of empirical studies about the effect of the COVID-19 pandemic on grief of bereaved relatives. As a result of the COVID-19 pandemic, deaths are characterized by poor bereavement outcomes and implications for mental and spiritual health. However, relatives have also showed signs of resilience in coping with their grief and attributing meaning to the loss of a loved one under stressful circumstances. Future research might compare the effect of the COVID-19 pandemic in (sub)cultures, to provide more detail on how cultural and societal differences relate to grief and the risks of grief disorders during COVID-19, in order to inform health care and social systems to optimize the support of patients, relatives, health care providers and the diversity of social communities.
